# Ameliorative potential of *Alstonia scholaris* (Linn.) R. Br. against chronic constriction injury-induced neuropathic pain in rats

**DOI:** 10.1186/s12906-017-1577-7

**Published:** 2017-01-19

**Authors:** Hasandeep Singh, Rohit Arora, Saroj Arora, Balbir Singh

**Affiliations:** 1 Department of Pharmaceutical Sciences, Guru Nanak Dev University, Amritsar, 143005 India; 2 Department of Botanical and Environmental Sciences, Guru Nanak Dev University, Amritsar, 143005 India

## Abstract

**Background:**

*Alstonia scholaris* commonly known as ‘Saptaparni’ is an Indian traditional medicinal plant used in Ayurveda. It is commonly used to treat various disorders like asthma, bronchitis, diarrhea, dysentery and malaria. In folklore medicine the milky juice of the plant is applied on wounds and ulcers to treat pain, ear ache and also in rheumatic pains.

**Aim:**

The present study was designed to investigate the potential of *A. scholaris* R. Br. in chronic constriction injury of sciatic nerve (CCI) induced neuropathic pain in rats.

**Methods:**

Peripheral neuropathy was induced by chronic constriction injury of sciatic nerve. The behavioral parameters like mechanical and thermal hyperalgesia and cold allodynia were assessed on the 14^th^ day. Tissue parameters like total protein, thiobarbituric acid reactive substances, reduced glutathione, myeloperoxidase, total calcium and TNF-α were assessed to check biochemical changes. Chloroform and methanol extract of *A. scholaris* leaves (100 and 200 mg/kg) and pregabalin (10 mg/kg, as positive control) were administered orally for 14 consecutive days starting from the day of surgery.

**Results:**

CCI resulted in significant development of mechanical hyperalgesia, heat hyperalgesia and cold allodynia along with alteration in the biochemical changes. Administration of methanol extract at 200 mg/kg significantly attenuated the CCI induced change in nociceptive threshold and biochemical changes which was comparable to that of pregabalin. The high-performance liquid chromatography (HPLC) of the bioactive methanol extract revealed the presence of different types of flavonoids such as gallic acid, catechin, epicatechin, ellagic acid and kaempferol, in which kaempferol was observed to be in higher concentration.

**Conclusion:**

Methanol extract (200 mg/kg) of *A. scholaris* showed the ameliorative effect in CCI induced neuropathic pain which may be due to the presence of kaempferol and attributed to its anti-oxidative and anti-inflammatory properties.

## Background

Neuropathic pain as stated by International Association for the Study of Pain is a pain generated by an abrasion or dysfunction in the nervous system [1, 2]. Generally, an abnormal sensation (dysesthesia), enhanced response to noxious stimuli (hyperalgesia) and pain due to hypo responsive stimuli (allodynia) are characteristics of neuropathic pain [3]. Patients with diabetes, AIDS, cancer, stroke and spinal cord injury are noted to demonstrate peripheral neuropathic pain [4]. Although, current drugs available for the effective management of neuropathic pain such as tricyclic antidepressants, antiepileptic drugs, cannabinoid receptor agonists and sodium channel blockers are effective, but, their usage is associated with many side effects [5]. On the other hand, traditional medicines provide a sigh of relief due to their unprecedented biological activities with least adverse effects.

Among an array of traditional plants, *Alstonia scholaris* (Linn.) R. Br. (Family Apocyanaceae), popularly known as “Saptaparni” in Hindi or the ‘Indian devil tree’ has been used in folk medicines for treating diarrhea, dysentery, malaria, fever and cardiac as well as respiratory problems [6, 7]. In folklore medicine, the milky juice of plant is applied on injuries and ulcers to treat pain including rheumatic pains [7]. Moreover, the plant afforded protection in various models of algesia and inflammation, including, acetic acid-induced writhing, formalin test and air pouch model in rodents [8]. Most of these pharmacotherapeutic effects of *A. scholaris* have been attributed to the presence of various phytoconstituents such as alkaloids, coumarins, iridoids, flavonoids, leucoanthocyanines, steroids, tannins, phenolics and saponins [9]. As traditional reports show that plant is useful in treating the normal and rheumatic pains and according to pharmacological reports the plant also exhibits anti-inflammatory and analgesic activities. Therefore, the plant *A. scholaris* was evaluated against neuropathic pain using rat model.

## Methods

### Plant material

Fresh leaves of *A. scholaris* were collected in September from the botanical garden, Guru Nanak Dev University, GNDU, Amritsar. Taxonomic identification of plant was done by Department of Botanical and Environmental Sciences, GNDU, Amritsar (voucher specimen No. 520). The leaves of *A. scholaris* were shade dried and reduced to coarse powder.

### Drugs and chemicals

One,1,3,3 tetra methoxy propane (Sigma Aldrich, Bangalore, India), reduced glutathione (GSH) (Loba Chemie, Mumbai, India) were used in the present study. Tumor necrosis factor-α (TNF-α) assay kit was procured from RayBiotech, Norcross, USA and total protein kit was procured from Span Diagnostics Ltd., Gujrat, India. Other reagents used in the present study were of analytical grade.

### Extraction

Soxhlet extraction of powdered plant material was carried out using different solvents with increasing order of polarity *viz* petroleum ether, chloroform and methanol. The obtained extracts were air-dried at room temperature to evaporate the solvent. Finally, the aqueous extract was obtained by digesting marc with distilled water for 24 h. Each extract was concentrated under pressure using rotatory evaporator (IKA Works Inc., North America). Dried concentrated extracts were finally weighed and their percentage yield was calculated. The final product was stored at 4 °C.

### Experimental animals

For animal studies and reporting, the authors adhered to guidelines of Committee for the Purpose of Control and Supervision of Experiments on Animals (CPCSEA), Ministry of Environment and Forest, Government of India, and ARRIVE guidelines. In present study, Wistar rats having body weight 250–300 g (procured from Indian Institute of Integrated Medicine, Jammu) were employed. Institutional Animal Ethics Committee of GNDU, Amritsar duly approved the animal experiments (Approval No. 226/CPCSEA/2013/07). Free access to rodent feed, water and necessary living environment was ensured in central animal facility of GNDU.

### Induction of peripheral neuropathy by chronic constriction injury (CCI)

Standardized model of chronic constriction injury-induced peripheral neuropathy was employed in present study [10]. In brief, rats were anaesthetized using chloral hydrate at 300 mg/kg and common sciatic nerve in left hind paw was exposed. Four loose ligatures of 4-0 chromic gut with 1 mm spacing were placed around the sciatic nerve. The muscle and skin were sutured in two separate layers. Sham surgery involved the exposure of sciatic nerve without inducing any lesion. The sterile conditions were ensured during surgery. All the treatments were given after CCI and animals survived for 2 weeks post-surgery.

### Experimental protocol

Forty-eight (48) animals were randomly assigned into 8 groups, each comprising 6 rats. The extracts were suspended in 0.5% carboxymethyl cellulose and were administered orally in rats.

- Group I (Normal control)
  No surgery was performed in rats.
- Group II (Sham control)
  Rats were subjected to surgical procedure to expose common sciatic nerve without inducing any injury to the nerve.
- Group III (CCI control)
  Rats were subjected to CCI to induce peripheral neuropathic pain.
- Group IV (Pregabalin) Standard drug
  Rats with CCI were treated with Pregabalin (10 mg/kg, oral) for 14 consecutive days post-surgery.
- Group V-VI (Chloroform extract (CE), 100 and 200 mg/kg)
  After sciatic nerve injury, the chloroform extract (100 and 200 mg/kg, oral) was administered in rats for 14 days.
- Group VII-VIII (Methanol extract (ME), 100 and 200 mg/kg)
  Methanol extract of *A. scholaris* (100 and 200 mg/kg, oral) was administered for 14 consecutive days in rats with CCI.

### Behavioral examination

- Assessment of mechanical hyperalgesia
  Pin prick test was employed to assess mechanical hyperalgesia in rats as described earlier [11]. In rats, the sharp end of bent needle was touched on lateral plantar surface of both hind paws with an intensity just sufficient to produce a reflex withdrawal. The paw withdrawal duration was recorded using a stopwatch with cut-off time of 20 s.
- Assessment of thermal hyperalgesia
  Thermal nociceptive threshold, an index of thermal-hyperalgesia was measured using hot plate method [12]. Rats were placed on Eddy’s hot plate (52 ± 1 °C) and withdrawal latency of paw (licking of the paw), was recorded. A cut-off time of 20 s was selected.
- Assessment of cold allodynia
  The reactivity towards chemical induced cold stimulus was determined by using established methods in rodents [13]. Briefly, the rats were placed on wired mesh grid, acetone was applied on plantar surface of their hind paw and withdrawal response was measured (in seconds). A cut-off time of 60 s was used in this protocol.

### Biochemical estimations

After 2 weeks of surgery, the rats were euthanized by cervical dislocation. The sciatic nerve tissue was excised and homogenized (10% w/v) using 0.1 M Tris buffer (pH-7.4). The contents were centrifuged (2000 × *g*) at 4 °C for 10 min. The clear supernatant so obtained was used for quantification of TNF-α, thiobarbituric acid reactive substances (TBARS), reduced glutathione (GSH) and total calcium content. Tissue surrounding the sciatic nerve was homogenized, followed by centrifugation at 5000 × *g* for 10 min. The pellet obtained was used for quantification of myeloperoxidase activity.

#### Estimation of TNF-α

Enzyme-linked immunosorbent assay based kit was used to estimate TNF-α levels in nerve homogenate. Total protein in nerve tissue was quantified using commercially available kit. Level of TNF-α was reported as pg/mg of protein.

#### Estimation of thio-barbituric acid reactive substances (TBARS)

The lipid peroxides in nerve tissue were quantified in terms of TBARS using established method [14]. The absorbance was measured spectrophotometrically at 532 nm. Results were expressed as nanomoles per mg of protein.

#### Estimation of GSH

GSH level in tissue was determined using method of Beutler et al. [15]. The GSH level was expressed as micrograms of reduced glutathione per mg of protein.

#### Estimation of total calcium

Total calcium level in sciatic nerve was measured using atomic absorption spectrophotometer as detailed by Severnghaus and Ferrebee [16]. Total calcium level was expressed as parts per million per mg of protein.

#### Estimation of myeloperoxidase (MPO) activity

The MPO activity was measured using method described by Krawisz et al. [17]. The MPO activity was expressed as units per gram of tissue.

### Chromatographic analysis

The TLC based fingerprinting profile of bioactive compounds in methanol extract was carried out using a precoated plates (Merck, Germany). Chloroform and methanol were used as solvent system in 99:1 ratio. Further, the methanol extract (1 mg/ml) was subjected to HPLC analysis. HPLC machine was fitted with RP-18 column (250 mm × 4 mm) 5 μm, an efficient degasser and a photodiode array detector (PDA) (Shimadzu Analytical (India) Pvt. Ltd.). Gradient elution was carried out using methanol and 0.1% acetic acid at a flow rate of 1 ml/min.

### Statistical analysis

The behavioral parameters (nonparametric data) were analyzed using Kruskal-Wallis test followed by Dunn's post hoc test. Data for biochemical estimations were analyzed using one-way analysis of variance followed by Tukey’s post-hoc test (Sigma stat version- 3.5 software). All the values were expressed as mean ± standard error of mean (S.E.M.). *P* ≤ 0.05 was considered to be statistically significant.

## Results

There was no significant change observed between control and sham groups. Therefore, all the comparisons were made with respect to control group.

### Effect of chloroform and methanol extract of *A. scholaris* on mechanical hyperalgesia in CCI induced neuropathy

**Fig. 1:**
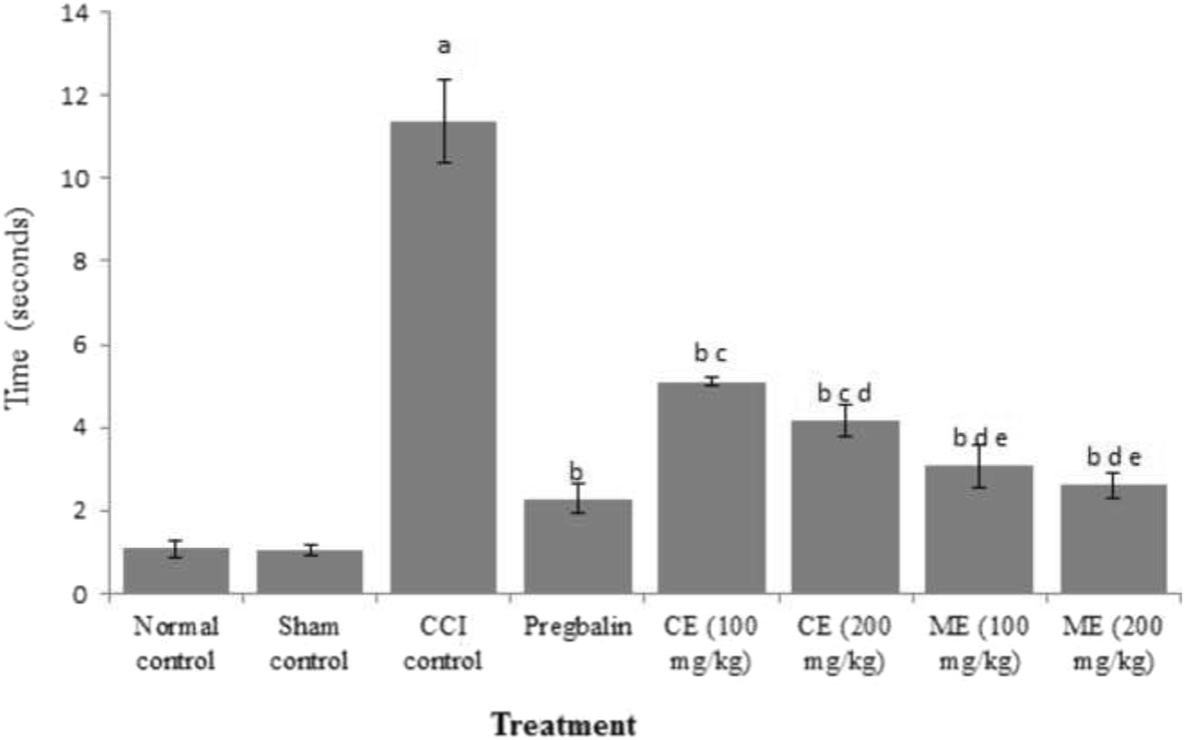
Effect of chloroform and methanol extracts on mechanical hyperalgesia in chronic constriction injury induced neuropathy. Values are expressed as mean ± S.E.M. a = *p* < 0.05 Vs control; b = *p* < 0.05 Vs CCI; c = *p* < 0.05 Vs pregabalin; d = *p* < 0.05 Vs chloroform extract (100 mg/kg); e = *p* < 0.05 Vs chloroform extract (200 mg/kg)

CCI led to mechanical hyperalgesia demonstrated by the significant increase in withdrawal threshold of hind paw in pinprick test as compared to control rats. Treatment of rats with pregabalin significantly ameliorated the paw withdrawal reflex. Administration of chloroform and methanol extracts of *A. scholaris* (100 and 200 mg/kg) markedly attenuated the CCI-induced increase in nociceptive threshold. Methanol extract exhibited better protection than chloroform extract against CCI-induced neuropathy in rats (Fig. 1).

### Effect of chloroform and methanol extract of *A. scholaris* on thermal hyperalgesia and cold allodynia in CCI-induced neuropathy

**Fig. 2:**
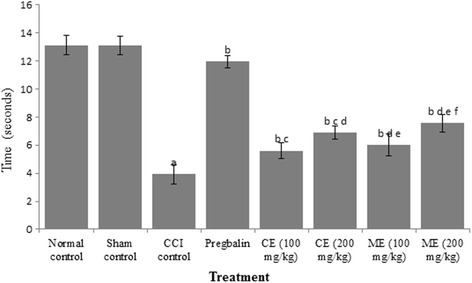
Effect of chloroform and methanol extracts on heat hyperalgesia in chronic constriction injury induced neuropathy. Values are expressed as mean ± S.E.M. a = *p* < 0.05 Vs control; b = *p* < 0.05 Vs CCI; c = *p* < 0.05 Vs pregabalin; d = *p* < 0.05 Vs chloroform extract (100 mg/kg); e = *p* < 0.05 Vs chloroform extract (200 mg/kg)

**Fig. 3:**
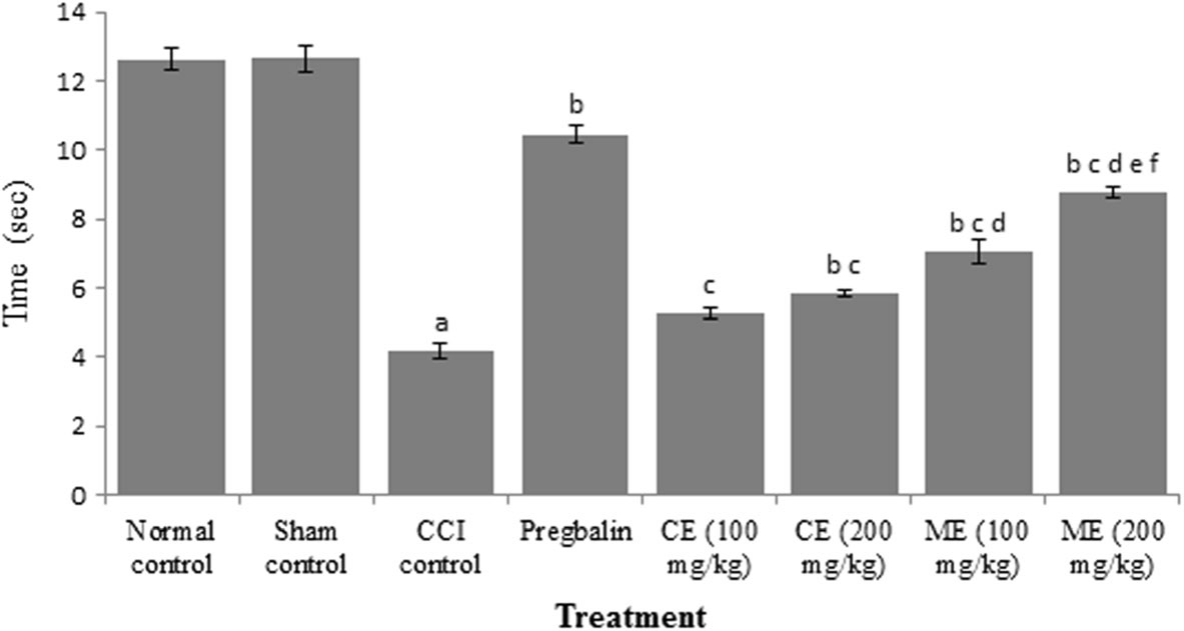
Effect of chloroform and methanol extracts on cold allodynia in chronic constriction injury induced neuropathy. Values are expressed as mean ± S.E.M. a = *p* < 0.05 Vs control; b = *p* < 0.05 Vs CCI; c = *p* < 0.05 Vs pregabalin; d = *p* < 0.05 Vs chloroform extract (100 mg/kg); e = *p* < 0.05 Vs chloroform extract (200 mg/kg); f = *p* < 0.05 Vs Methanol extract (100 mg/kg)

The constriction of sciatic nerve resulted in a significant thermal hyperalgesia and cold allodynia, which was ameliorated by pregabalin treatment in rats. Administration of chloroform and methanol extracts (100 and 200 mg/kg) attenuated the CCI-induced reduction in nociceptive threshold for thermal hyperalgesia and cold allodynia (Figs. 2 and 3).

### Effect of chloroform and methanol extract of *A. scholaris* on oxidative stress markers

**Fig. 4:**
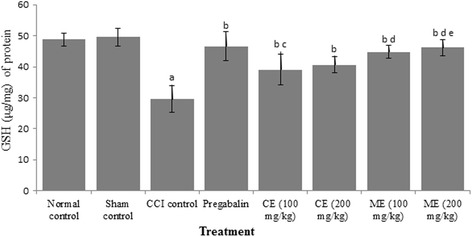
Effect of chloroform and methanol extracts on reduced glutathione activity in chronic constriction injury induced neuropathy. Values are expressed as mean ± S.E.M. a = *p* < 0.05 Vs control; b = *p* < 0.05 Vs CCI; c = *p* < 0.05 Vs pregabalin; d = *p* < 0.05 Vs chloroform extract (100 mg/kg); e = *p* < 0.05 Vs chloroform extract (200 mg/kg)

**Fig. 5:**
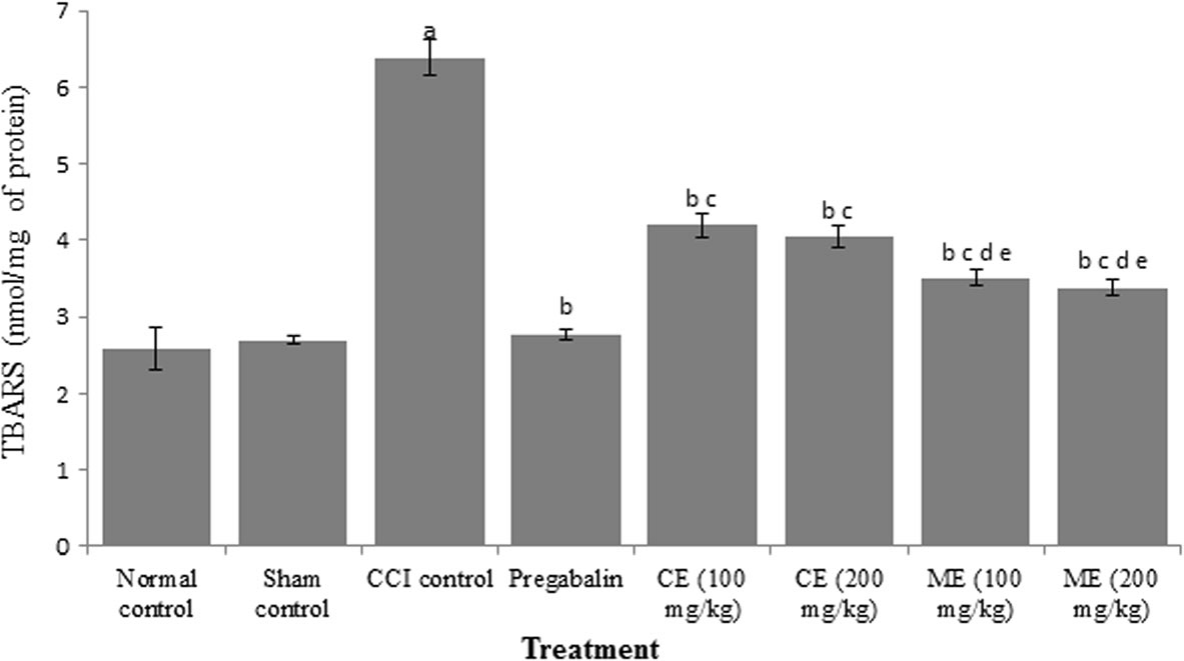
Effect of chloroform and methanol extracts on thio-barbituric acid reactive substances (TBARS) in chronic constriction injury induced neuropathy Values are expressed as mean ± S.E.M. a = *p* < 0.05 Vs control; b = *p* < 0.05 Vs CCI; c = *p* < 0.05 Vs pregabalin; d = *p* < 0.05 Vs chloroform extract (100 mg/kg); e = *p* < 0.05 Vs chloroform extract (200 mg/kg)

Increase in lipid peroxides (measured in terms of TBARS and decrease in GSH) levels was observed in CCI group as compared to control group. Pregabalin significantly ameliorated the oxidative stress in rats. Treatment of animals with chloroform and methanol extract of *A. scholaris* (100 and 200 mg/kg) attenuated CCI-induced increase in TBARS and decrease in GSH levels. Moreover, the methanol extract afforded better protection than chloroform extract in rats. (Figs. 4 and 5).

### Effect of chloroform and methanol extract of *A. scholaris* on total calcium and inflammatory markers in CCI-induced neuropathy

**Fig. 6:**
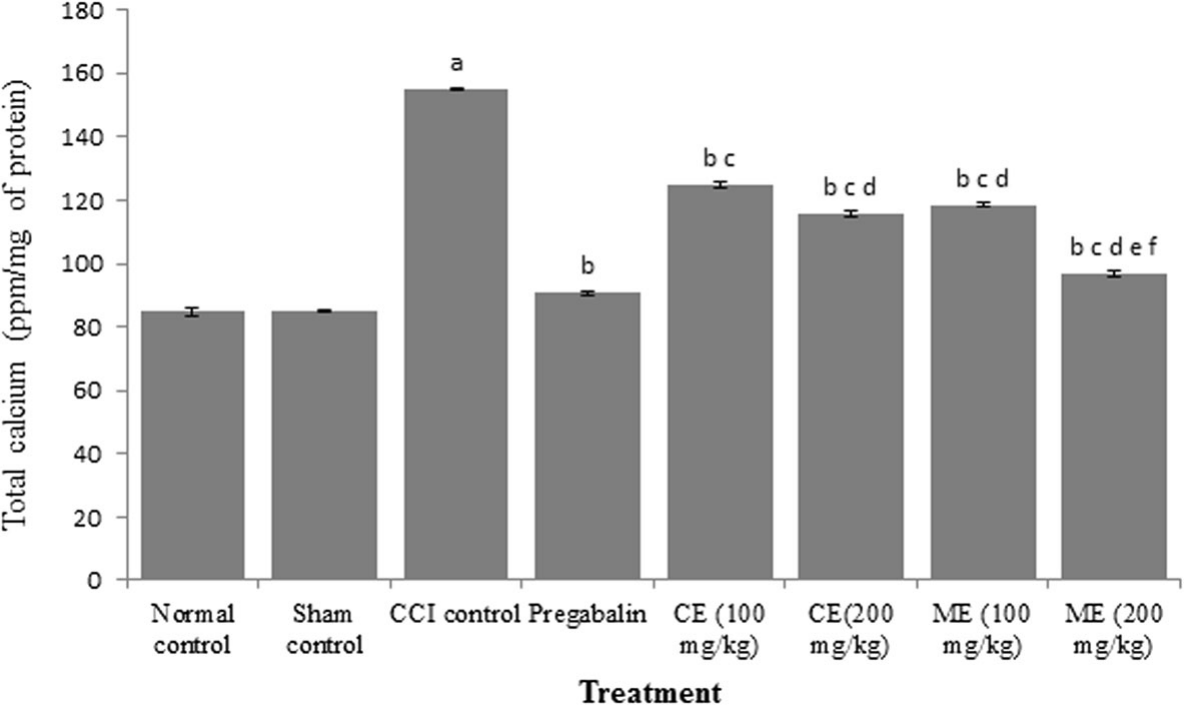
Effect of chloroform and methanol extracts on calcium levels in chronic constriction injury induced neuropathy. Values are expressed as mean ± S.E.M. a = *p* < 0.05 Vs control; b = *p* < 0.05 Vs CCI; c = *p* < 0.05 Vs pregabalin; d = *p* < 0.05 Vs chloroform extract (100 mg/kg); e = *p* < 0.05 Vs chloroform extract (200 mg/kg); f = *p* < 0.05 Vs Methanol extract (100 mg/kg)

**Fig. 7:**
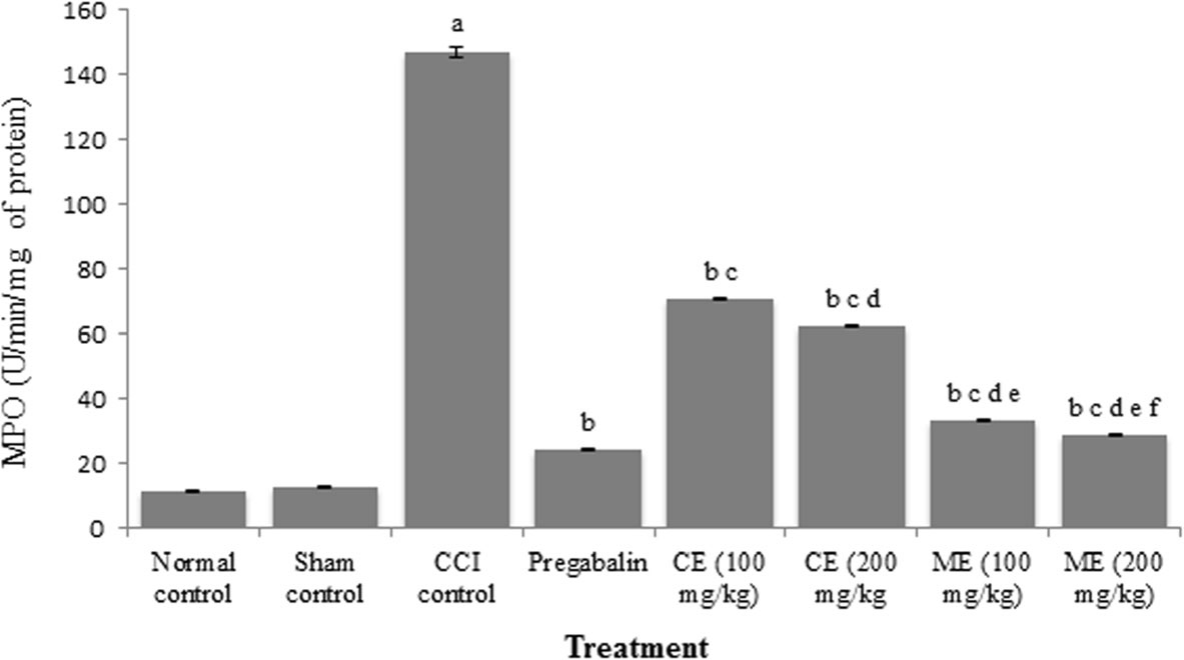
Effect of chloroform and methanol extracts on myeloperoxidase activity in chronic constriction injury induced neuropathy. Values are expressed as mean ± S.E.M. a = *p* < 0.05 Vs control; b = *p* < 0.05 Vs CCI; c = *p* < 0.05 Vs pregabalin; d = *p* < 0.05 Vs chloroform extract (100 mg/kg); e = *p* < 0.05 Vs chloroform extract (200 mg/kg); f = *p* < 0.05 Vs Methanol extract (100 mg/kg)

**Fig. 8:**
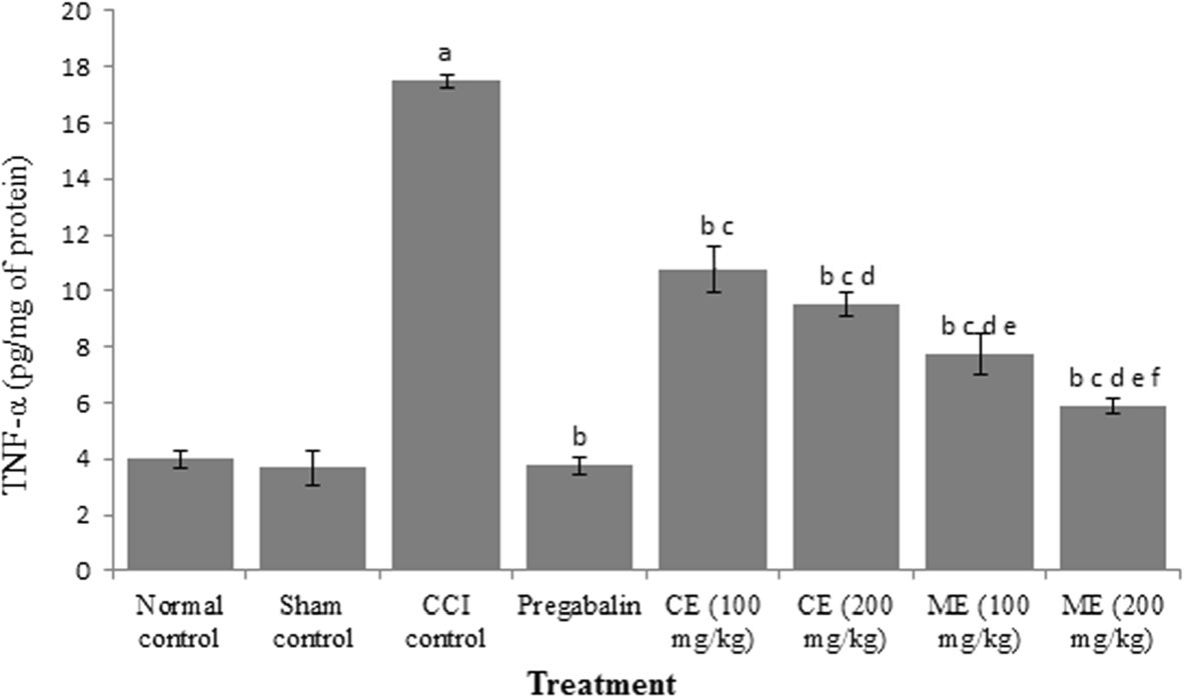
Effect of chloroform and methanol extracts on TNF-α levels in chronic constriction injury induced neuropathy. Values are expressed as mean ± S.E.M. a = *p* < 0.05 Vs control; b = *p* < 0.05 Vs CCI; c = *p* < 0.05 Vs pregabalin; d = *p* < 0.05 Vs chloroform extract (100 mg/kg); e = *p* < 0.05 Vs chloroform extract (200 mg/kg); f = *p* < 0.05 Vs Methanol extract (100 mg/kg)

CCI resulted in a significant increase in total calcium, TNF-α and myeloperoxidase (MPO) in sciatic nerve of CCI rats as compared to control group. Pregabalin treatment markedly reduced total calcium and inflammatory cytokines. Administration of chloroform and methanol extract (100 and 200 mg/kg) obviated CCI induced increase in total calcium, TNF-α and myeloperoxidase activity. Treatment with methanol extract witnessed better anti-inflammatory activity than chloroform extract in rats. (Figs. 6, 7 and 8).

### Chromatographic analysis

The TLC based fingerprinting profile of bioactive constituents in methanol extract showed the presence of 5 spots (Table 1). The results of high performance liquid chromatogram of methanol extract are shown in Table 2.

**Table 1:** TLC fingerprinting profile of methanol extract of *A. scholaris* using chloroform and methanol as mobile phase (99:1). The values indicate the R_f_ values of the separated bands

Sr. No. of Resolving Bands	UV 254 nm	UV 366	Under day light
1.	–	–	0.25
2.	–	–	0.34
3.	–	–	0.75
4.	–	–	0.87
5.	–	–	0.93

**Table 2:** Compounds revealed in high performance liquid chromatography at different retention time

Peak#	Ret. Time	Area	Heigh	Conc.	Unit	Mark	Name
1.	1.894	125,395	4092	0.000			
2.	2.733	16,307	812	1.144	mg/L	V	Gallic acid
3.	3.559	1715	114	0.718	mg/L		Catechin
4.	5.673	1636	151	0.430	mg/L		Epicatechin
5.	15.684	135,192	4469	29.733	mg/L		Ellagic acid
6.	15.887	49,079	4773	0.000		V	
7.	16.204	266,914	5061	42.677	mg/L	V	
8.	17.224	235,946	3191	100.534	mg/L	V	Kaempferol
Total		832,184	22,663				

## Discussion

Chronic constriction induced sciatic nerve injury is one of the widely-employed models to evaluate potential agents for the management of neuropathic pain in rodents [9]. The CCI-induced neuropathic pain is observed in various clinical situations such as arthroplasty, complex regional pain syndrome, fracture as well as stroke [18]. Various cellular events such as alteration in the calcium homeostasis, release of pro-inflammatory mediators (TNF-α and MPO) and generation of reactive oxygen species lead to neuronal damage and neuropathic pain [19, 20]. The sustained activation of peripheral nociceptive receptor leads to hypersensitization of secondary neurons and central nervous system [21].

In the present investigation, the ameliorative effect of *A. scholaris* in CCI induced neuropathic pain had been observed. CCI resulted in mechanical hyperalgesia, thermal hyperalgesia and cold allodynia demonstrated by pin prick method, hot plate method and acetone drop test, respectively. The change in pain threshold in the said models indicated successful induction of peripheral neuropathy in rats. It is documented that these behavioral alterations start on 3^rd^ day after surgery and attain their peak in 2 weeks [22]. The chronic treatment with chloroform and methanol extracts of *A. scholaris* (100 and 200 mg/kg) for 14 days significantly increased nociceptive threshold as observed with mechanical hyperalgesia, heat hyperalgesia and cold allodynia test. However, *A. scholaris* methanol extract (200 mg/kg) have been observed to have better pharmacotherapeutic effects in comparison to chloroform extract for management of CCI induced neuropathic pain.

Increased levels of reactive oxygen species (ROS) in the body cause cell damage or cell death. Therefore, removal of excessive ROS is important to restore normal conditions. It is a proven fact that ROS are one of the major perpetrators in the induction and progression of neuropathic pain [23]. Marked increase in TBARS and decrease in GSH level in sciatic nerve demonstrated oxidative stress in CCI group, which is in accordance with previous findings. Treatment with chloroform and methanol extracts (100 and 200 mg/kg) for 14 days resulted in marked attenuation of neuropathic pain and oxidative stress in animals. However, methanol extract at 200 mg/kg showed better protection against oxidative stress. Our results are supported by the fact that other free radical scavengers such as phenyl-N-tert-butylnitrone showed profound reduction of spinal nerve ligation-induced mechanical allodynia in rats. Furthermore, spin trap agents like 5,5-dimethylpyrroline-N-oxide and nitroso benzene demonstrated similar effects thereby highlighting the detrimental role in the development of neuropathic pain [24].

We observed a significant increase in inflammatory cytokine including TNF-α and myeloperoxidase activity in sciatic nerve. The CCI-induced neuropathy has also been associated with significant inflammation as assessed by marked increase in MPO and TNF-α levels. The neutrophils are known to release MPO and rise in MPO activity is a well-defined marker for inflammation [25]. Various research groups have demonstrated a significant association between oxidative stress and inflammation in CCI-induced neuropathic pain [26, 27]. It is reported that inflammatory mediators such as bradykinin, prostaglandins, TNF-α, interleukin-1β are involved in induction as well as progression of neuropathic pain [28, 29]. Calcium ion accumulation has been well documented to play an important role in post-traumatic axotomy, CCI, anti HIV drugs and vincristine induced neuropathy [30–32]. Another interesting finding of the study was restored calcium accumulation in the sciatic nerve in animals having CCI induced neuropathic pain. Muthuraman and Singh have reported significant increase in total calcium in sciatic nerve in CCI group than the control group. Similar results are witnessed in our studies. Intracellular accumulation of calcium triggers various biochemical changes including hyper-excitability, activation of phospholipases, proteolytic enzymes. These biochemical changes result in cellular damage and death [33, 34]. In the neuronal tissues, calcium induced activation of calpains has been reported to degrade axonal cytoskeleton and thus resulting in axonal degeneration [35]. Administration of chloroform and methanol extracts (100 and 200 mg/kg) for 14 days resulted in marked attenuation of inflammatory cytokines and calcium levels. Thus, decrease calcium level may be another molecular mechanism which may explain the ameliorative effect of *A. scholaris* on for the management of CCI-induced neuropathic pain. Interestingly methanol extract of *A. scholaris* at 200 mg/kg showed better antioxidant and anti-inflammatory activity than chloroform extract. On the same lines administration of methanol extract attenuated neuropathic pain better than chloroform extract. Our results suggest that antioxidant and anti-inflammatory activity of the methanol extract of *A. scholaris* accounts for its neuroprotective activity.

Flavonoids are secondary plant metabolites and possess significant antioxidant activity. Various flavonoids such as myrcetin, rutin, quercetin and kaempferol are noted to relieve neuropathic pain induced by various culprit agents [36–43]. Antioxidant potential of *A. scholaris* pertaining to presence of various polyphenols such as gallic acid, catechin, epicatechin, ellagic acid and kaempferol may explain its significant ameliorative effect in CCI induced neuropathic pain. Thus, significant antioxidant potential of *A. scholaris* might be responsible for improvement in behavioral, and biochemical outcomes in CCI induced neuropathic pain in rats.

However, further studies are required to consolidate our findings using other models of neuropathic pain.

## Conclusion

The present study concluded that *A. scholaris* substantially ameliorated CCI induced neuropathic pain in rats. *A. scholaris* treatment reduced calcium deposition in nerve, which could be the major mechanism responsible for its neuroprotective potential besides inhibiting the inflammatory cytokines and ROS production.

## Abbreviations

%: Percentage
°C: Degree centigrade
ANOVA: Analysis of variance
CCI: Chronic Constriction Injury
cm: Centimeter
CMC: Carboxymethylcellulose
CNS: Central nervous system
DTNB: 5, 5’-Dithio, bis (2-nitro benzoic acd)
g: Gram
GSH: Glutathione
HETAB: Hexadecyl Trimethyl Ammonium Bromide
*i.p*: Intraperitoneal
IASP: International Association for the study of Pain
mg: Milligram
ml: Millilitre
MPO: Myeloperoxidase
nmol: Nanomole
*p.o*.: Per oral
S.E.M: Standard error of mean
sec: Second
μl: Microlitre
